# Serum LDH, a prognostic factor in elderly patients with acute myelogenous leukaemia

**DOI:** 10.1054/bjoc.2000.1537

**Published:** 2001-01-01

**Authors:** C D Dalley, T A Lister, J D Cavenagh, A Z S Rohatiner

**Affiliations:** ICRF Medical Oncology Unit, St Bartholomew's Hospital, London, EC1A 7BE, United Kingdom


British Journal of Cancer (2001) **84**(1), 147
© 2001 Cancer Research Campaign
doi: 10.1054/ bjoc.2000.1537, available online at http://www.idealibrary.com on IDEAL®

Letter to the Editor

### Serum LDH, a prognostic factor in elderly patients with acute myelogenous leukaemia

Sir,

We read with interest the recent letter correlating pre-induction serum LDH to clinical outcome in patients with germ cell tumours by Shamash et al (2000), and would like to confirm the findings of Ånström and co-workers (2000) with regard to serum LDH and clinical outcome in older patients with acute myelogenous leukaemia (AML).

Between July 1987 and December 1999 124 consecutive patients with newly diagnosed AML (age > 60 years) were managed at St Bartholomew's Hospital. 75 patients were selected for treatment with curative intent on clinical grounds, and received mitoxantrone in combination with cytarabine (mitoxantrone 12 mg/m$^2$ i.v. for 3 days; $n$ = 49, 10 mg/m$^2$ i.v. for 5 days; $n$ = 26, cytarabine 100 mg/m$^2$ bd i.v. 7 days). The remaining patients received supportive care with blood products and antibiotic therapy as appropriate.

Complete remission (CR) was achieved in 34/75 patients (45%) (27% of the entire cohort). The only factor predictive of CR on multivariate analysis was age. With a median follow up of 5.5 years, for patients treated with curative intent, actuarial survival at 1, 3 and 5 years was 33%, 10% and 7% respectively. By univariate analysis, serum LDH, cytogenetic subgroup and patient age correlated with overall survival. 'Unfavourable' karyotype and raised LDH (>twice upper limit of normal) were found to be the factors most highly predictive for poor overall survival by multivariate analysis (Table 1).

The clinical outcome for older patients with AML is frequently disappointing. Many of those treated with curative intent experience significant morbidity, in addition, 30% die as a result of complications associated with induction therapy, and the CR rate is often no better than 40–50% (Hiddemann et al, 1999). Thus the decision to offer treatment with curative intent to the older person with AML is not straightforward.

**Table 1** Prognostic factors for overall survival in newly diagnosed elderly patients treated with mitoxantrone and cytarabine

| Multivariate analysis | HR | 95% confidence interval | *P* value |
|---|---|---|---|
| Serum LDH* (>twice upper limit of normal) | 3.29 | 1.38–7.86 | *P* = 0.01 |
| Age/(5 year increment) | 1.84 | 1.15–2.95 | *P* = 0.01 |
| Cytogenetic sub-group[a] (base line: Normal) | | | |
| 'Unfavourable' | 4.24 | 1.42–12.6 | *P* = 0.01 |

HR>1 indicates poor overall survival. Serum LDH*: normal range (240–480 IU/l). Cytogenetic sub-group[a]: 'Unfavourable' sub-group [-5/5q-, -7, Complex karyotype (3 or more distinct chromosomal rearrangements)].

Serum LDH is an important prognostic factor, predicting for clinical outcome in both haematological and non-haematological malignancy. By utilizing this simple laboratory test, alone or in combination with other prognostic factors, it may be possible to select older patients who are likely to benefit from curative therapy (Ferrara and Mirto, 1996).

*CD Dalley, TA Lister, JD Cavenagh and AZS Rohatiner*
*ICRF Medical Oncology Unit, St Bartholomew's Hospital, London, EC1A 7BE, United Kingdom*

### REFERENCES

Ånström M, Bodin L, Nilsson I and Tidefelt U (2000) Treatment, long-term outcome and prognostic variables in 214 unselected AML patients in Sweden. *Br J Cancer* **82**: 1387–1392

Ferrara F and Mirto S (1996) Serum LDH value as a predictor of clinical outcome in acute myelogenous leukaemia of the elderly. *Br J Haematol* **92**: 627–631

Hiddemann W, Kern W, Schoch C, Fonatsch C, Heinecke A, Wormann B and Buchner T (1999) Management of acute myeloid leukaemia in elderly patients. *J Clin Oncol* **17**: 3569–3576

Shamash J, Oliver R, Gallagher C, Newland A, Lister T, Kelsey S, Gupta R and O'Doherty C (2000) Pre-induction LDH as a prognostic factor for outcome of high dose chemotherapy (HDCT) for germ cell tumours relapsing or refractory to conventional chemotherapy. *Br J Cancer* **82**: 2022–2023

